# Viruses and Extracellular Vesicles: Special Issue, 2020, with Thirteen Articles by Chioma M. Okeoma

**DOI:** 10.3390/v12111265

**Published:** 2020-11-06

**Authors:** Chioma M. Okeoma

**Affiliations:** Department of Pharmacology, Stony Brook University Renaissance School of Medicine, Stony Brook, NY 11794-8651, USA; chioma.okeoma@stonybrook.edu; Tel.: +1-(631)-444-3014

The discovery of extracellular vesicles (EVs) dates back to the early 1940s, when Erwin Chargaff and Randolph West showed that platelet-free plasma contains coagulation components that pellet upon high-speed (31,000× *g*) centrifugation [1]. Subsequently, other investigators made similar findings, including the 1967 publication on “platelet dust” by Peter Wolf [2] and the 1970 finding by Webber and Johnson that platelet alpha granules are associated with vesicles [3]. These and other pioneering studies laid the foundation for the EV field. By definition, EV is a generic name that describes a heterogeneous collection of mostly circulating membranous vesicles. The term EV, although inclusive of subgroups, such as ectosomes, exosomes, microvesicles, microparticles, oncosomes, and prostasomes, is often used interchangeably in the field. EVs are released by all cell types from the three domains of life—Archaea, Bacteria, and Eukarya. As a result, EVs are an integral part of life and they regulate biological activities including but not limited to inter-cellular communication, lateral/horizontal gene transfer, and response to stimuli and infectious agents, such as bacteria, fungi, parasites, and viruses. 

Of interest is the similarities between viruses and EVs [4]. Virus-infected cells produce EVs, which in turn may render uninfected cells susceptible to infections. On the other hand, EVs isolated from some biological fluids display antiviral functions or serve as decoys to neutralize viruses and limit their replication. Given the similarities between viruses and EVs, along with the tremendous research effort in the EV and virology fields, we organized this Special Issue (SI) “Viruses and Extracellular Vesicles” to deliver both high-impact review and primary research articles to national and international EV researchers. This SI provides researchers information regarding progress in EV biology and the intersection with virology. In this first edition of the SI, https://www.mdpi.com/journal/viruses/special_issues/Viruses_EVs, a diversity of investigators through their articles reflect on ongoing research as well as the future of EVs in viral pathogenesis and therapeutics. 

In their research article, Kaddour et al. used body fluid EVs isolated from the blood and semen of HIV infected and HIV uninfected men to provide biophysical and biological information on the role of surface electrostatic properties of EVs [5]. The study highlights the effect that HIV infection has on blood and semen EVs while revealing tissue-specific differences in surface electrostatic properties of EVs. The study further reveals the critical role sialic acids-associated EVs play on the surface charge and EV internalization by target cells [5]. The interaction between HIV and EVs was further investigated by Barclay et al. The group used HIV-1 latently infected cell lines to uncover a potential mechanism behind EV-mediated activation of latent HIV-1. They showed that EV-associated c-Src activated EGFR/PI3K/AKT/mTOR/STAT3/SRC-1 signal, resulting in HIV-1 latency reversal [6]. The focus on EV cargo was also addressed by Grabowska et al., who showed that the EV-associated envelope glycoprotein B (gB) homologs of various Alpha herpesviruses differentially alter MHC Class II molecules and serve as a potential immunoregulatory mechanism of viral gB proteins [7]. Further analysis of the association of EVs with viruses was provided by Iša et al., with the observation that rotavirus particles are associated with EVs and that this association may promote rotavirus infection [8]. Together, the studies from the Okeoma [5], Kashanchi [6], Lipińska [7], and Arias [8] groups used clinical samples or cultured cell lines to address the crosstalk between EV-associated viral and host proteins from a basic virology perspective.

In their study, Ishikawa et al. used transcriptome analysis to reveal the mRNA profile of milk-derived EVs isolated from Bovine Leukemia Virus-Infected Cattle [9]. McGowan et al., in their pilot study, evaluated the enrichment profile of EV-associated miRNAs from the blood of HEV- and HCV-infected patients [10]. These transcriptomic studies from the Inoshima [9] and Petrik [10] groups are great resources to the EV community as they provide information on the RNA biotypes associated with EVs in different mammalian body fluids under different disease conditions. 

In addition to the primary research articles above, excellent reviews by different investigators summarize a wide array of studies on functional effects of EVs and mechanistic insights regulating EV-mediated roles. The review by Alqatawni et al., from the Daniel group, focused on the role of EVs in HIV infection and wound healing. The group summarized published evidence of the involvement of EVs in coagulation, inflammation, proliferation, and extracellular matrix remodeling, all of which are processes involved in wound healing that may be applicable to HIV and other viruses [11]. Insights into the role of EVs in viral replication, pathogenesis, antiviral response, and therapeutic interventions were summarized by Kumar et al., from the Santosh Kumar group [12], and Bello-Morales et al., from the López-Guerrero group [13]. The review by Reyes-Ruiz et al. from the del Ángel group focused on the potential role of EVs in flavivirus dissemination and transmission from the insect vector to host cells [14]. These reviews describe current knowledge about the involvement of EVs in viral pathogenesis and highlight the potential of EVs in the treatment of viral infections. 

Additionally, Simone Giannecchini summarized published evidence on the mechanisms by which polyomaviruses (PyVs) exploit the EV delivery system during infection [15]. The investigator proposes that the association of PyV miRNAs with EVs in body fluids may be suggestive of a potential PyV persistence. In their review, Giannessi et al. discussed the state of the art of the studies on the relationship between EVs and various viruses including HIV, HCV, and SARS viruses [16]. This review from the Affabris group [16] placed the potential involvement of EVs in the pathogenesis of SARS viruses in a historical framework with the knowledge of well-known EV–HIV and EV–HCV interactions. To cap this edition of SI, Kutchy et al., from the Buch group, used the interaction between EVs and HIV, HTLV, Zika, CMV, EBV, HepB, HepC, JCV, and HSV to discuss the potential roles of EVs in virus-mediated neurodegenerative diseases and how EVs and their cargos may serve as biomarkers and therapeutic vehicles for viral infections [17]. 

Combining all the studies published in this SI edition, a theme that echoes the involvement of EVs in the pathogenesis of various viruses emerges. Also evident is the usefulness of EVs in the development of biomarkers and therapeutic strategies against various viruses. 

I would like to end by acknowledging everyone that contributed to the success of this SI—including all the authors, editors, reviewers, as well as scientists and researchers of primary articles in our reviews. I am encouraging the academic, scientific, and medical communities to join the virology and EV communities in reading these articles.
